# Editorial: Harnessing artificial intelligence in sports science: enhancing performance, health, and education

**DOI:** 10.3389/fspor.2026.1780876

**Published:** 2026-03-05

**Authors:** José Eduardo Teixeira, Pedro Forte, Ricardo Ferraz, Ryland Morgans, Luís Branquinho

**Affiliations:** 1 Department of Sports Sciences, Polytechnic of Guarda, Guarda, Portugal; 2 Department of Sports Sciences, Polytechnic of Cávado and Ave, Guimarães, Portugal; 3 SPRINT—Sport Physical Activity and Health Research & Innovation Center, Guarda, Portugal; 4 CI-ISCE, Instituto Superior de Ciências Educativas do Douro (ISCE Douro), Penafiel, Portugal; 5 Department of Sports Sciences, Polytechnic Institute of Bragança, Bragança, Portugal; 6 Research Center for Active Living and Wellbeing (LiveWell), Polytechnic Institute of Bragança, Bragança, Portugal; 7 Department of Sport Sciences, University of Beira Interior, Covilhã, Portugal; 8 Research Center in Sports, Health and Human Development, Covilhã, Portugal; 9 School of Sport and Health Sciences, Cardiff Metropolitan University, Cardiff, United Kingdom; 10 Biosciences Higher School of Elvas, Polytechnic Institute of Portalegre, Portalegre, Portugal; 11 Life Quality Research Center (LQRC-CIEQV), Santarém, Portugal

**Keywords:** AI, big data, deep learning, health, machine learning, physical education, sports performance

Artificial intelligence (AI) has emerged as a transformative force within sports science, enabling unprecedented advances in the understanding, monitoring, and optimization of human performance, health, and learning processes. The increasing availability of high-resolution data derived from wearable technologies, tracking systems, video analysis, and digital learning platforms has necessitated the adoption of advanced analytical frameworks capable of managing complexity, non-linearity, and inter-individual variability. Within this context, the present research topic brings together eleven contributions that collectively demonstrate how AI-driven methodologies can enhance performance analysis, support health-oriented decision-making, and foster innovation in sports performance, health, and education.

A prominent focus across several contributions is the application of machine learning techniques to performance profiling and training optimization. Foucaud et al. apply an unsupervised learning approach to identify recovery profiles in elite canoe-kayak athletes across an Olympic season. Their findings illustrate how data-driven clustering methods can reveal latent recovery patterns that are not readily observable through traditional analytical approaches, supporting individualized monitoring strategies in high-performance environments. Similarly, Barsumyan et al. utilize machine learning models to analyze cardiovascular drift during prolonged cycling exercise, offering practical insights into physiological regulation under sustained load and reinforcing the value of AI for endurance performance assessment.

Predictive modeling also emerges as a key theme. Cordeiro et al. propose a synthetic data-based machine learning framework to predict performance attenuation, addressing common limitations associated with small sample sizes in elite sport. This approach represents an important methodological advancement, highlighting how artificial data augmentation can enhance model robustness while preserving ecological validity. Such tools have clear implications for load management, injury prevention, and long-term athlete development.

Beyond physiological modeling, the Research Topic emphasizes the growing relevance of deep learning and computer vision in sports science. Chen et al. provide a comprehensive review of computer vision applications in sport, quantitatively mapping technological evolution since the early 21st century. Their synthesis underscores the rapid expansion of automated motion analysis, tactical evaluation, and performance feedback systems, while also identifying persistent challenges related to accuracy, generalization, and contextual interpretation. These findings reinforce the need for interdisciplinary collaboration between sport scientists, engineers, and data scientists.

The interpretability of AI models is another critical concern addressed in this collection. Iapteff et al. introduce Bayesian mixed models for expected goals analysis, demonstrating how probabilistic frameworks can enhance transparency and uncertainty estimation in performance analytics. This contribution aligns with growing calls for explainable AI, particularly in applied sport contexts where decisionmakers require both accuracy and interpretability to support tactical and strategic choices. Importantly, the research topic also addresses ethical and methodological challenges associated with AI deployment. O’Mara and Mahmud critically examine grading bias in machine and deep learning applications within rock climbing, raising important questions regarding fairness, representation, and model generalizability. Their work serves as a timely reminder that technological progress must be accompanied by rigorous evaluation of bias and equity, particularly as AI-based assessment tools gain prominence in sport and education.

Health promotion and injury prevention represent a complementary pillar of the research topic. Li explores the application of deep learning models in public health-oriented physical training, highlighting AI's potential to support exercise prescription, monitoring, and long-term health outcomes. Tan et al. extend this perspective by examining AI-informed optimization of physical education scheduling, emphasizing the role of intelligent systems in promoting sustainable participation and population-level health benefits. Together, these studies illustrate how AI can bridge elite performance science and public health objectives.

In addition to the contributions discussed above, the systematic review by Teixeira et al. provides a comprehensive synthesis of how AI is used to map tactical behavior and collective dynamics in football. This review collates evidence from multiple studies using neural networks, machine learning, and deep learning approaches applied to spatiotemporal tracking data and tactical performance indicators, revealing how AI models can uncover latent patterns of team formation, space control efficiency, and player interactions. Importantly, the study highlights applications that extend from real-time decision support for coaches to the design of training tasks that enhance overall team cohesion and collective execution. Despite these advances, the authors note ongoing challenges related to model interpretability, integration into operational coaching contexts, and the need for interdisciplinary expertise that blends sport science with advanced analytics.

Complementing, Xie et al. conducted a quasi-experimental study to enhance table tennis performance and physical fitness through a “3 + 1” digital teaching model grounded in the Technological Pedagogical Content Knowledge (TPACK) framework. Integrating multimedia, virtual reality, augmented reality, and AI, this instructional model was tested on university students and demonstrated improvements in both sport-specific skills and general physical conditioning. The findings support the potential of blended technological and pedagogical frameworks to elevate the effectiveness of sport education, particularly by leveraging immersive and intelligent technologies to deliver personalized and engaging learning experiences.

Educational innovation constitutes a further dimension of this research topic. Gao highlights the integration of AI within higher education physical education, emphasizing personalized learning pathways, adaptive feedback, and intelligent evaluation systems. By aligning technological innovation with pedagogical frameworks, this work demonstrates how AI can enhance student engagement, skill acquisition, and lifelong participation in physical activity.

Collectively, the eleven articles within this research topic illustrate the multifaceted impact of AI across sports science. They demonstrate that AI is not merely a tool for automation or prediction but a catalyst for conceptual and methodological innovation. From sports elite performance optimization to health promotion and educational transformation, these contributions highlight the importance of responsible, interpretable, and context-aware AI systems. In conclusion, this research topic provides a comprehensive snapshot of current advances and emerging directions in AI-driven sports science research. By integrating computational intelligence with domain-specific expertise, the studies presented here lay a strong foundation for future interdisciplinary research and applied innovation. Continued efforts to balance technological sophistication with ethical responsibility and practical relevance will be essential to fully harness the potential of AI in sports science.

